# How physical activity enhances academic engagement in middle school: a serial mediation model of interpersonal relationships and academic support

**DOI:** 10.3389/fpsyg.2025.1632130

**Published:** 2025-08-11

**Authors:** Junmin Zhang, Yongfeng Liu, Xiaogang Zhang, Shuiyou Hu

**Affiliations:** ^1^School of Sports Training, Chengdu Sport University, Chengdu, China; ^2^School of Football Sports, Chengdu Sport University, Chengdu, China; ^3^School of Physical Education, Chengdu Sport University, Chengdu, China

**Keywords:** academic engagement, physical activity, interpersonal relationships, academic support, serial mediating model

## Abstract

**Introduction:**

Academic engagement is a critical aspect of adolescent development. Understanding the mechanism of academic engagement will help to take effective measures to improve the academic engagement of middle school students. This study used a serial mediation model to delve into the complex association among physical activity, interpersonal relationships, academic support, and academic engagement in middle school students.

**Methods:**

A sample of 746 participants in middle school (51.1% female) completed validated measurement tools for each variable.

**Results:**

Results showed that grade was negatively correlated with physical activity and academic engagement, yet positively associated with interpersonal relationships, and significant positive correlations among the four variables. In contrast, gender and only-child status had weak correlations with other measured variables. Findings from regression analyses further underscored that physical activity positively directly predicted academic engagement. Notably, mediation analyses showed that both interpersonal relationships and academic support mediated the separate relationship between physical activity and academic engagement, and they also supported the hypothesized serial mediation effect. The final serial mediation model effectively explained a substantial 31.8% portion of the variance in academic engagement.

**Discussion:**

These findings comprehensively illustrate the complex interactions between physical activity and academic engagement, deepening our understanding of the determinants of the relationship between the two variables among middle school students. The study provides valuable empirical evidence for designing targeted interventions aimed at enhancing adolescents’ academic engagement through improved physical activity, interpersonal relationships, and academic support.

## 1 Introduction

In recent decades, educational psychology has witnessed growing interest in the concept of “engagement” as a proper antonym to burnout. Schaufeli and Bakker (2004) outlined engagement as a positive occupational state of mind characterized by vigor, dedication, and absorption. Applied to the educational environment, academic engagement defines active participation of students in formal education activities, such as the fulfillment of coursework and involvement in learning exercises (Johnson and Johnson, 2009). Research has consistently demonstrated that academic motivation is a strong predictor of short-term academic success (Johnson and Johnson, 2009; Hershberger and Jones, 2018) and future functional growth (Fredricks et al., 2016; Abid and Akhtar, 2020) from middle school (Kwon, 2020) through high school contexts (Qudsyi et al., 2020). Existing literature demonstrated that high academic engagement enhances academic achievement (Johnson and Sinatra, 2013), and another study discovered a significant correlation between academic engagement and better physical and mental health conditions (Wefald and Downey, 2009). In addition, researchers emphasized academic engagement contribution to school adaptation of students (Wang and Fredricks, 2014). While research has proven that low academic engagement of students is at increased risk of academic underachievement, school dropout, drug use, delinquency, and adverse emotional states such as anxiety and depression (Leslie et al., 2010; Li and Lerner, 2011). These findings point to the need for additional research on academic engagement and the implementation of effective interventions to promote student engagement in school.

### 1.1 Physical activity and academic engagement

Growing evidence suggests that physical activity, which is defined as bodily movement produced by skeletal muscles that expends energy, is associated with a range of health benefits and specifically can increase academic engagement through psychosocial and neurocognitive mechanisms (Owen et al., 2016). Researchers have addressed the relationship between physical activity and academic engagement from diverse perspectives. On the one hand, a meta-analysis of the positive correlation between physical fitness, a significant byproduct of regular physical activity, and academic achievement indicates that active students tend to perform better academically (Castelli et al., 2007). Moreover, one study investigated the acute cognitive effects of physical activity, demonstrating that brief bouts of exercise can enhance attention, memory, and executive functioning, which are crucial for academic participation (Tomporowski et al., 2011).

On the other hand, focused interventions might trigger scholarly involvement. Doolittle researched structured physical activity programming in schools to demonstrate their potential to enhance students’ enthusiasm for learning and classroom participation (Doolittle, 2016). Similarly, researchers analyzed resource utilization strategies for promoting physical activity, identifying key facilitators that increase students’ physical and academic engagement (Wang et al., 2023). Classroom research has discovered that the integration of physical activity breaks into the curriculum improves students’ concentration, motivation, and overall participation in the learning process (Spring et al., 2023). Notably, some evidence reported that chronic physical exercise is linked to long-term cognitive gains from longitudinal measurement (Lambourne and Tomporowski, 2010) and proved the high correlation between physical activity and engagement, specifically in obese children (McCoy and Rupp, 2021) validating the long-term benefit of chronic exercise for academic performance, especially in vulnerable populations. Despite these advances, critical gaps persist in the literature. The mechanisms through which physical activity influences academic engagement across development are limited, especially among middle school students undergoing fast physical, cognitive, and social changes. Thus, we propose the following hypothesis:

*H1*: Physical activity positively predicts academic engagement.

### 1.2 Interpersonal relationships, physical activity, and academic engagement

Interpersonal relationships—such as with family, peers, romantic partners, and community members—are a fundamental part of human life that has a significant influence on behavior, health, and well-being across the lifespan. Klem and Connell (2004) first proposed the paramount position of interpersonal relationships in school engagement. Since then, research has investigated the relationship between interpersonal relationships and personal aspirations (Collie et al., 2016). Most informative are peer relationship studies (Shao et al., 2024), flipped classroom settings (Li, 2021), teacher-student, and peer relationship interactions (Košir and Jugović, 2025). Specifically, peer relationships are impactful in fostering feelings of belonging and cooperation for middle school students (Wentzel, 1991).

There is robust evidence demonstrating a bidirectional relationship between physical activity and social relationships. Evidence indicates that physical activity enhances interpersonal relationships through the direct construction of self-confidence and social competence, resulting in better peer relations (Spring et al., 2023). Physical activity is a buffer against stress for university students, increasing the quality of relationships and overall psychological health (Joo, 2018). Physical activity improves social skills in teens, particularly in key transitional periods such as middle school (Hu and Tan, 2025). The above indicates not just the promotion of health behavior through physical activity but also stress reduction, enhanced self-efficacy, and improved social skills at various stages of life.

Additionally, empirical evidence consistently highlights the pivotal role played by interpersonal relationships in academic engagement. Interpersonal relationships between teachers and students, and peers are direct motivators of engagement (Collie et al., 2016), and indirect influences through psychological mediators such as personal best goals and self-efficacy (Benlahcene et al., 2024). These relationships manifest distinct roles in social contexts: teacher interactions facilitate cognitive engagement through intellectual challenge and feedback, peer relationships facilitate emotional engagement by satisfying belonging needs, and family relationships offer instrumental support that removes practical barriers to learning (Zhu et al., 2025). During critical developmental phases, the teacher assumes a relational amplifier role, augmenting the positive impact of peer interactions on engagement by satisfying students’ fundamental need for relatedness (Košir and Jugović, 2025). Combined, these findings exhibit interpersonal relationships that promote academic interaction through multiple channels. Thus, we hypothesize the following:

*H2*: Interpersonal relationships will mediate the relationship between physical activity and academic engagement.

### 1.3 Academic support, interpersonal relationships, physical activity, and academic engagement

Interpersonal relationships serve as the foundational framework through which social support influences health behaviors, as articulated by social support theory (Baron et al., 1990). Instrumental support reduces practical barriers to physical activity, and parental modeling of active lifestyles and joint activities (e.g., family walks, cycling) correlate with higher physical activity levels in adolescents (Sallis et al., 2015). Emotional support addresses psychological barriers like lack of confidence or isolation; spouse support among the elderly, for example, ensures prolonged physical activity maintenance by inspiring opportunity for group exercise, which encourages motivation and reduces seclusion (FioRito and Routledge, 2020). Through peer networks that provide informational support and encouragement for physical activity, adolescents are encouraged to participate in moderate-to-vigorous activity to attain group membership (Martins et al., 2021).

Support from teachers is particularly important in fulfilling students’ fundamental psychological needs for relatedness and competence (Klem and Connell, 2004), encouraging engagement directly while facilitating achievement indirectly (Tao et al., 2022). This teacher-student relationship functions with other origins of critical support: parents have valuable structural and instrumental resources to offer, as well as good emotional support and cooperative learning provision (Chen, 2005; Qudsyi et al., 2020). These relational supports indicate that teacher scaffolding is particularly essential for underachievers, whereas high achievers are more likely to be helped by peer-based learning relations (Moreira et al., 2018). These findings highlight the multidimensional nature of academic support systems, with different relationships playing different and interdependent roles in promoting student achievement.

Evidence of the relationship between support and physical activity shows age-dependent patterns and contextual variations across populations. In later life, social support is essential to facilitate physical activity by removing some primary barriers, such as social isolation, mobility issues, and motivational deficits (Lindsay Smith et al., 2017). In contrast, peer support is the predominant predictor of adolescent physical activity participation, especially for team sport and vigorous activity, which attests to the developmental importance of social belonging and identity formation during this life stage (Prochaska et al., 2002; Mendonça et al., 2014). Meta-analytic findings specifically reveal a moderate positive association between peer support and physical activity in adolescent girls, with peer influence outperforming parental support effects consistently, while these relations demonstrate cultural and methodological variability (Laird et al., 2016). These general research findings reveal the relationship between support and physical activity. The elderly influence physical activity by support, but in adolescents, there is an association, and the direction of causality is uncertain. Probably, a bidirectional influence exists between them.

Besides interpersonal relationships, other research shows that a wide support system can enhance student engagement and academic achievement through unique and complementary processes. Landmark research put in place the foundation of knowledge of academic support systems as an aggregate process of enabling student achievement (Chen, 2005). Subsequent research has all examined these processes, demonstrating the way school-based support fits with academic achievement (Moreira et al., 2018) and operates dynamically through day-to-day interactions (Robayo-Tamayo et al., 2020). Meta-analytic estimates (Tao et al., 2022) have subsequently measured these associations, with concurrent studies on social support constructs (Jayarathna, 2015; Gutiérrez et al., 2017) further elucidating their role in explaining variance in student engagement (Wilcox et al., 2016). These initial studies collectively emphasize support systems as multifaceted networks that extend beyond interpersonal relationships to include structural and institutional levels. Strong interpersonal relationships, forged through physical activity, cultivate a network of trust that enables students to effectively seek and receive academic support—whether from peers, teachers, or parents—by reducing perceived barriers to support-seeking and enhancing the perceived reliability of assistance.

The emerging theoretical framework comprises a series of initial findings. Physical activity is association with social competence (Doolittle, 2016) and academic resource utilization (Wang et al., 2023) has been well-documented, as have the bidirectional relationships between relational quality and support systems (Robayo-Tamayo et al., 2020). Additional insights come from studies examining parent–child communication (Geng et al., 2023) and the interplay between self-esteem and engagement (Zhao et al., 2021), collectively contributing to a comprehensive understanding of academic engagement determinants.

Based on the relationships between variables identified in the above literature and using relevant scales that can measure variables, we explored variables that may be associated with academic engagement, including physical activity, interpersonal relationships, and academic support, which can measure academic resources and different sources of support, interpersonal relationships and academic support are related to other variables among them, and hypothesized that there is a mediation effect between the four variables. Interpersonal relationships and academic support were hypothesized to be mediating variables, playing a sequential mediating role in the physical activity and academic engagement of middle school students. This research addresses critical gaps in existing literature by unraveling the cognitive mechanisms through which physical activity influences academic engagement among middle school students. Specifically, we move beyond documenting mere correlational associations to explore how social interactions and psychological resources sequentially mediate this relationship. Furthermore, the findings offer tangible implications for educational practice, including the design of school - based intervention programs, formulation of evidence - informed policies, and development of parental support strategies. These dual contributions—advancing theoretical understanding of adolescent learning processes and providing actionable insights for real - world application—highlight the study’s interdisciplinary significance.

Within this model framework, we proposed the following hypotheses:

*H3*: Academic support mediates the relationship between physical activity and academic engagement.

*H4*: A serial multiple mediation model whereby physical activity influences academic engagement through the sequential mediating pathways of interpersonal relationships and academic support, structured as physical activity → interpersonal relationships → academic support → academic engagement.

**Figure 1:**
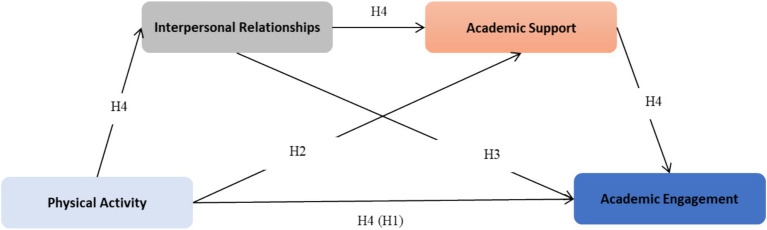
The framework for hypotheses.

All hypotheses are shown in Figure 1.

## 2 Methods

### 2.1 Participants

Before the research, the participants were adequately trained to complete the test with a complete understanding of the requirements and instructions. Cluster sampling was employed in the study, where middle school students from two schools in Sichuan Province, China, were sampled. Having obtained informed consent from schools, teachers, and parents, the students completed the survey anonymously. Written informed consent was elicited from each participant, and they were informed of their right to refuse to participate.

760 students aged between 13 and 17 years were involved in the survey, out of which 746 participants (96.84%) provided valid information. 365 participants were male (48.9%), and 381 were female (51.1%). Participants were divided grade-wise as follows: 161 students of seventh standard (21.6%), 154 students of the eighth standard (20.6%), 144 students of the ninth standard (18.8%), 145 students of tenth standard (19.4%), and 146 students of eleventh standard (19.6%).

The Chengdu Sports University Research Ethics Committee permitted the current research (NO. CTYLL2025004). Procedures were carried out according to proper guidelines and rules and in harmony with the Declaration of Helsinki on ensuring the safety of human participants in research. Written informed consent was voluntarily given by all the participants and their parents.

### 2.2 Measurements

#### 2.2.1 Physical activity rating scale

The physical activity rating scale (PARS-3) developed by Liang and Liu (1994) was used to assess the exercise levels with three dimensions: intensity (1–5 scale), frequency (1–5 scale), and duration (0–4 scale). The total activity score was calculated as “intensity × frequency × duration” (range: 0–100). The Cronbach’s *α* of the scale was determined to be 0.703 in the current study.

#### 2.2.2 Interpersonal relation synthetic diagnose test

The interpersonal relation synthetic diagnose test compiled by Zheng (1999) contains 28 dichotomous items (1 = yes, 0 = no) measuring four dimensions: communication distress, social interaction distress, interpersonal relationship distress, and heterosexual interaction distress. Higher total scores indicate greater interpersonal difficulty, and lower levels of interpersonal relationships. While swapping the values 0 and 1 for high scores indicates great interpersonal relationships. This test measures difficulties in communication, was selected because it operationalizes the absence of healthy relationship functioning, which we argue is conceptually inverse to relationship quality. The Chinese version has established reliability in adolescent populations (Liu, 2021) with a total scale α of 0.873 in this study.

#### 2.2.3 Academic support scale for middle school

The academic support scale for middle school (Zheng et al., 2024) utilized a 5-point Likert scale (1 = strongly disagree, 5 = strongly agree) to measure four dimensions: emotional support, willingness support, resource support, and behavioral support. The whole scale showed excellent reliability (α = 0.952) in the present study.

#### 2.2.4 Academic engagement questionnaire

The Chinese version of the academic engagement questionnaire (Li and Huang, 2010), adapted from Schaufeli’s UWES-S (Schaufeli et al., 2002) contains 17 items rated on a 7-point Likert scale (1 = never, 7 = always). It has three dimensions: motivation, energy, and concentration, with excellent reliability (α = 0.968) in this study.

### 2.3 Statistical analysis

The present study employed a double-platform analysis approach using RStudio (Version 4.4.2; R Core Team, 2024) and IBM SPSS Statistics (Version 27.0) and the results from both software tools were fully congruent to ensure the robustness of analysis and verification of findings. The main analysis was conducted using the BruceR package (Bao, 2024), which is a fast statistical package designed for behavioral and social science studies. Integrated functionality offered by BruceR touches every phase of the research cycle, starting from high-level data management capabilities like clever data import/export operations in different file formats to variable recoding procedures that consume less memory. The psychometric analysis feature within the package simplifies scale scoring through coding routines as well as reliability checking. To deal with more sophisticated statistical modeling, BruceR incorporates an easy-to-use extension of Hayes’ (Hayes, 2018) PROCESS procedure for moderation and mediation analysis with bootstrap estimation of confidence intervals - a feature that is particularly helpful in testing complex theoretical models. The package also shines with an automated reporting process producing publication-quality output and visual tools incorporated.

Our analysis process involved two consecutive steps. The initial data preparation stage was typified by assertive data cleansing methods, including outlier identification and exclusion criteria application, followed by careful descriptive statistics to identify the underlying characteristics of our dataset and examine relationships. The second analytical phase, theoretically grounded in the current literature, aimed to test a serial mediation model to explain complicated relationships between our four key variables. By contemporary methodological convention (Hayes, 2018), we employed bias-corrected bootstrap estimation with 5,000 resamples to yield 95% confidence intervals for all indirect effects. The statistical significance criterion was that the confidence intervals did not contain zero at the conventional *α = 0.05* level.

### 2.4 Check for common method bias

To assess the possibility of common method variance (Aguirre-Urreta and Hu, 2019) in using only self-reports, we conducted a Harman single-factor test (Podsakoff et al., 2003). Exploratory factor analysis without rotation indicated that 13 distinct factors emerged from the test with a cumulative variance of 61.00%. Most significantly, the first principal component explained a mere 21.10% of variance, a far cry from the magic mark of 40%, which would indicate common method bias (Zhou and Long, 2004). The pattern of results thus suggests that common method bias is not a threat to the validity of our findings.

## 3 Results

### 3.1 Descriptive statistics and correlations

Table 1 presents the descriptive statistics for all study variables among our sample of middle school students (*N = 746*). Physical activity levels, as measured by the Physical Activity Rating Scale, were found to be moderate (*M = 43.82, SD = 22.65*) with a nearly normal distribution. Academic engagement yielded the highest mean score (*M = 86.01, SD = 22.71*), while interpersonal relationship difficulties showed moderate levels (*M = 18.77, SD = 6.13*). Academic support scores were positively skewed (*M = 81.66, SD = 16.58*), with generally high perceived support. Demographic variables gave measures, with grade levels being almost symmetrical (*M = 8.95, SD = 1.43*), genders almost an even split (51.10% female), and 71.60% singletons only child. All variables gave adequate parameters of normality (Skewness: −0.94 to 0.40; Kurtosis: −2.00 to 3.05), and there were no signs of floor or ceiling effects in measures.

**Table 1:** Descriptive statistics among variables.

Variables	Mean	SD	Median	Min	Max	Skewness	Kurtosis
Grade	8.95	1.43	9	7	11	0.05	−1.33
Gender	1.51	0.5	2	1	2	−0.04	−2.00
Only child	1.71	0.45	2	1	2	−0.94	−1.12
Physical activity	43.82	22.65	45	1	100	0.4	−0.24
Interpersonal relationships	18.77	6.13	19	0	28	−0.59	0.13
Academic support	81.66	16.58	81	27	135	0.28	3.05
Academic engagement	86.01	22.71	87	17	119	−0.71	0.54

**Figure 2:**
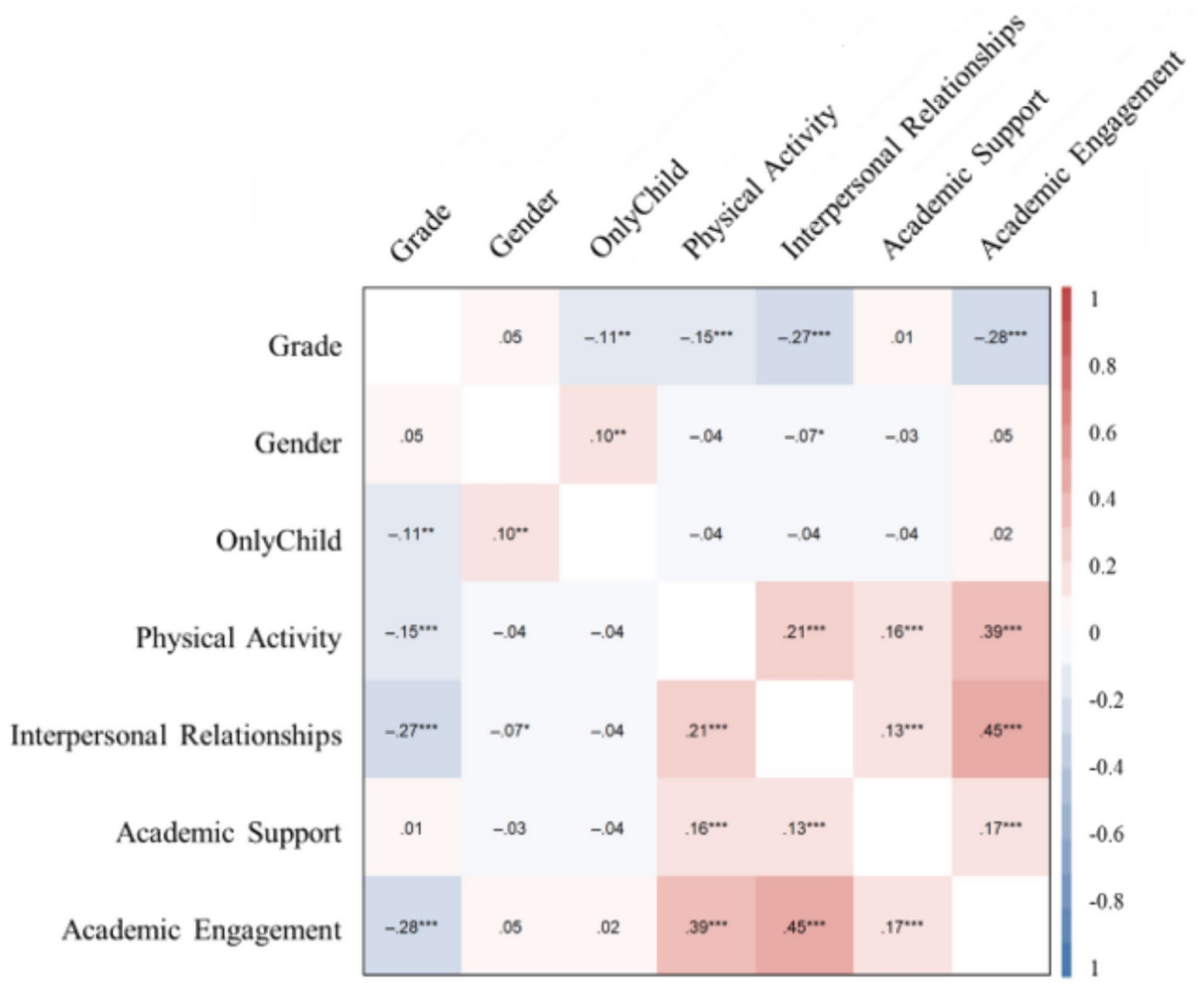
Correlation coefficient between variables.

The analysis revealed significant Pearson correlations among the variables shown in Figure 2. Several significant relationships emerged: (i) Grade level showed negative correlations with physical activity (*r = −0.15, p < 0.001*), interpersonal relationships (*r = −0.27, p < 0.001*), and academic engagement (*r = −0.28, p < 0.001*); (ii) Physical activity was positively correlated with interpersonal relationships (*r = 0.21, p < 0.001*), academic support (*r = 0.16, p < 0.001*), and academic engagement (*r = 0.39, p < 0.001*); (iii) Interpersonal relationships showed positive relationships with both academic support (*r = 0.13, p < 0.001*) and engagement (*r = 0.45, p < 0.001*); (iiii) Academic support was positively correlated with academic engagement (*r = 0.17, p < 0.001*).

Notably, the strongest correlation was found between interpersonal relationships and academic engagement (*r = 0.45*). In contrast, demographic variables such as gender and only child status were generally weakly associated with the other measured variables. These results highlight the complex interactions among grade, physical activity, interpersonal relationships, and academic factors, providing valuable insights into the phenomenon under study.

### 3.2 Testing for the serial mediation model

**Figure 3:**
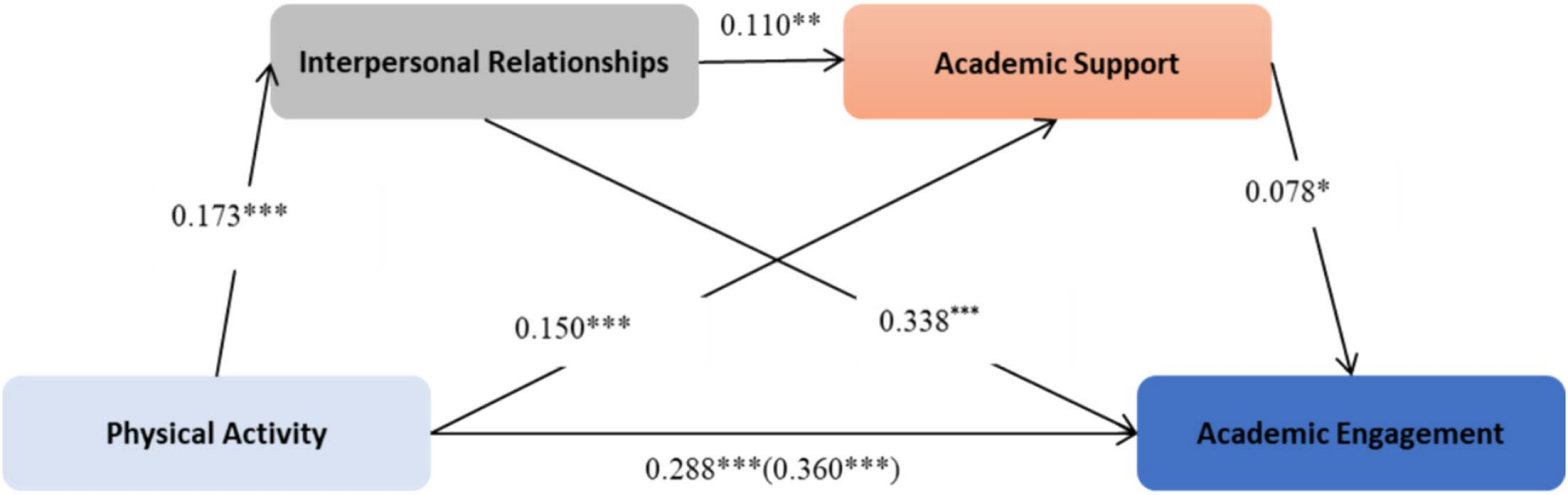
The serial mediation model.

The regression analysis (Table 2) yielded several key findings regarding the factors influencing academic engagement. Physical activity showed a strong positive effect on academic engagement (*β = 0.360, p < 0.001*), while interpersonal relationships demonstrated a substantial positive prediction with academic engagement (*β = 0.338, p < 0.001*). Academic support exhibited a modest positive effect on engagement (*β = 0.078, p < 0.05*). Grade level negatively predicted academic engagement (*β = −0.224, p < 0.01*) and interpersonal relationships (*β = −0.241, p < 0.001*) significantly, but positively predicted academic support (*β = 0.060, p > 0.05*). Notably, physical activity had a positive effect on interpersonal relationships (*β = 0.173, p < 0.001*), suggesting a potential protective effect. The model diagram of serial mediation is shown in Figure 3.

**Table 2:** Regression models among variables.

Variables	Model 1: academic engagement	Model 2: interpersonal relationships	Model 3: academic support	Model 4: academic engagement
Grade	−0.224***	−0.241***	0.060	−0.145***
	(0.033)	(0.035)	(0.038)	(0.032)
Physical activity	0.360***	0.173***	0.150***	0.288***
	(0.033)	(0.035)	(0.037)	(0.032)
Interpersonal relationships			0.110**	0.338***
			(0.038)	(0.032)
Academic support				0.078*
				(0.031)
*R* ^2^	0.204	0.101	0.039	0.317
Adj. *R*^2^	0.201	0.098	0.035	0.314

Standardized regression coefficients are displayed, with standard errors in parentheses. *p < 0.05, **p < 0.01, ***p < 0.001.

The final comprehensive regression model (Model 4) provided a robust explanation for the variance in academic engagement, accounting for 31.8% of it (*R^2^ = 0.317*). This outcome underscores the combined influence of physical activity, interpersonal relationships, academic support, and grade level in shaping academic engagement.

Bootstrap analysis indicated that the mediation effect was strong in the hypothesized model. The total indirect effect was statistically significant and accounted for roughly 20.28% of the total effect. Of the individual indirect paths, the mediation effect through interpersonal relationships was the strongest and accounted for roughly 16.54% of the total effect. The indirect effect through academic support and serial mediation of interpersonal problems and academic support was small, at 3.27% and 0.42%, respectively, but significant. Importantly, the direct effect was significant, accounting for 79.72% of the total effect (see Table 3).

**Table 3:** The bootstrapping analysis of the mediating effects.

Variables	Effect	BootSE	BootLLCI	BootULCI	Proportion
Indirect_all effect	0.072	0.015	0.044	0.102	20.28%
Indirect effect 1	0.059	0.014	0.034	0.089	16.54%
Indirect effect 2	0.012	0.006	0.002	0.023	3.27%
Indirect effect 3	0.001	0.001	0.000	0.004	0.42%
Direct effect	0.288	0.037	0.216	0.361	79.72%
Total effect	0.360	0.038	0.289	0.435	100.00%

SE and CI are estimated based on 1,000 Bootstrap samples. LL, low limit; CI, confidence interval; UL, upper limit. Indirect effect 1: Physical Activity_ Interpersonal relationships _Academic Engagement; Indirect effect 2: Physical Activity _Academic Support _Academic Engagement; Indirect effect 3: Physical Activity _Interpersonal relationships _Academic Support _Academic Engagement.

## 4 Discussion

### 4.1 Descriptive statistics and correlations

Our findings indicate several significant trends in adolescent development that need to be carefully monitored. The moderate physical activity levels observed are consistent with recent longitudinal studies (Guthold et al., 2020); similar decline is most likely indicative of increased scholastic burdens and shifting social priorities during this age bracket. The relatively high academic engagement scores, despite significant individual differences, are consistent with cross-cultural studies of East Asian education systems (Kitamura et al., 2022), in which academic achievement is highly prized, causing a situation of fewer social relationships. These are consistent with contemporary developmental theories because of the especially crucial social adjustment in early adolescence (Blakemore, 2019). Our results extend this by showing how physical activity can serve as a buffer against social adversity, in line with new neurodevelopmental evidence (Orben et al., 2020). Academically, our findings contribute to the mounting literature on school effects (Wang and Hofkens, 2020), especially in collectivist cultures where institutional support is key to student outcomes.

In correlational terms, the negative correlation between grade level and physical activity is consistent with recent multinational surveys (Aubert et al., 2022). While the strong correlation between physical activity, like exercise, and academic engagement validates emergent hypotheses of exercise-mediated neuroplasticity (Morris et al., 2020) and is consistent with emerging intervention trials in schools (Daly-Smith et al., 2021). The correlations of academic support may imply complex influences, which require additional examination using experience sampling (Larson et al., 2022).

### 4.2 Physical activity and academic engagement

This suggests the need for intervention at critical developmental stages to sustain motivation. The physical activity prediction of academic motivation is in keeping with Hypothesis H1, reinforcing earlier work (Sallis et al., 2015) that has identified physical activity as enhancing cognitive resources (e.g., attention, memory, executive function) and motivational states, and thus providing a stronger foundation for active learning. For instance, Donnelly et al. (2016) found that school physical activity interventions significantly improved on-task behavior and academic achievement, particularly in math and reading. These findings are in line with neuroscientific research (Hillman et al., 2014) that aerobic exercise increases prefrontal cortex activity and neurogenesis, both of which are critical for self-regulation and sustained attention during academic performance.

Given this strong evidence, this study underscores the importance of physical education as an effective means of strengthening academic involvement. Further support comes from a longitudinal study (Booth et al., 2014), in which adolescents engaging in regular physical activity had increased levels of academic attainment over time, which was mediated by increased concentration and reduced absenteeism. Policymakers and educators should thus prioritize physical activity for its health impact and as a key initiator of academic success.

### 4.3 The mediating role of interpersonal relationships

Evidence highlights that shared physical activity experiences, such as team sports and group exercises, act as powerful boosters for cooperation, trust, and social interaction (Blakemore, 2019), aligning with Hypothesis H2. Eime et al. (2013) demonstrated that participation in sports in an organized setting produces social capital by enhancing the peer network and an overwhelming feeling of belonging, both of which feature prominently in adolescent psychosocial development. More studies revealed that school-based physical activity programs reduce social isolation and enhance prosocial behavior among vulnerable youth by creating environments in which positive social norms are supported (Lubans et al., 2016). Moreover, longitudinal studies reveal that students whose peer relationships were developed through cooperative physical activities have increased classroom engagement and report increased teacher and peer academic support (Oberle et al., 2019). This aligns with social interdependence theory (Johnson and Johnson, 2009), which contends that cooperative environments—like team sports—develop mutual accountability, shared goals, and motivational spillover into academic domains. A meta-analysis showed that physically active adolescents develop stronger teacher-student relationships and social skills, processes statistically mediating increased academic achievement (Visier-Alfonso et al., 2021).

Together, these studies illuminate the psychosocial process by which exercise enhances academic engagement, secondarily augmenting the social scaffolding (interpersonal relationships, teacher support) that supports it. For this to be maximized, schools would emphasize collective physical activities, e.g., team sports, class athletic competitions, or collective fitness challenges, whose explicit aim is both physical health and interpersonal quality.

### 4.4 The mediating role of academic support

This model examines the impact of academic support, where physical activity and social relationships are the prime roles. Physical activity’s positive impact on academic support (by way of greater peer/teacher interactions) is consistent with social support theory (Baron et al., 1990), in which active students are able to gain more instrumental benefits (e.g., study skills guidance) and emotional support. This is consistent with a longitudinal study (Howie et al., 2018) because physical activity had minimal direct influences on school performance, but its influence was powerfully mediated through increased teacher support and peer interaction. Similarly, Michael et al. (2023) showed in an intervention school that engaged students established more supportive teacher-mentor relationships that enabled increased support-seeking behaviors regarding studies. The weak but positive pathway from physical activity to participation in school work through support structures copies evidence (Owen et al., 2016), showing school sport activity enhanced social capital, indirectly impacting school results by opening access to learning assets.

This model emphasizes the interlinked physical activity and relational determinants of support system development, providing support for Hypothesis H3. The linear link between physical activity and participation in academic activity, and augmented mediation through support systems, is supported by evidence, whose meta-analysis established that when physical activity’s academic effect was greatest, it was when mediated by psychosocial factors like teacher support and classroom belonging Vasilopoulos et al. (2023). This suggests that schools might be able to reap academic dividends on exercise through explicitly developing social-support mechanisms in which such dividend payments are achieved.

### 4.5 The serial mediation model

This model specifies one critical serial mediation path through which physical activity influences academic engagement: by improving interpersonal relationships first, which in turn raises perceived academic support, and ultimately enhancing academic involvement. This chained process is squarely within Hypothesis H4 and is rooted in theoretical and empirical foundations. The sequence begins with the well-documented contribution of physical activity to social relationships (Eime et al., 2013). This first step in mediation is further supported, and network analysis found that physically active students hold more central positions in classroom social networks, providing them greater access to academic resources (Janssen and Connelly, 2021). These positive relationships are then succeeded by actual academic support, as predicted by social support theory (Baron et al., 1990). The longitudinal findings powerfully speak to this second link - though physical activity had modest direct contributions to academic performance, its influence was largely mediated through increased teacher support and peer collaboration (Howie et al., 2018). The complete serial mediation pathway echoes the social-academic integration model proposed (Oberle et al., 2021), who demonstrated that peer acceptance mediated the effect of physical activity on classroom engagement through subsequent increases in teacher support. Notably, Parker et al. (2022) demonstrated that interventions explicitly designed to leverage this two-pathway pathway (e.g., cooperative PE paired with peer tutoring) resulted in academic competencies larger than activity-only programs.

Our serial mediation model generates major theoretical contributions. The interpersonal challenges pathway informs updated social competence theories (Durlak et al., 2022), showing how physical activity promotes prosocial behaviors via skills acquisition and offering opportunities (Doolittle, 2016). The academic support findings inform existing models of resilience (Martin et al., 2022), with physical activity being a potential stimulus for support utilization. The biopsychosocial model is supported by our evidence of parallel mechanisms (Smith et al., 2021). The serial mediation model emphasizes that the academic advantages of physical activity are not isolated but emerge from an active network of relationships and support. While physical activity exerts a robust direct influence, its impact is further shaped by social dynamics in educational settings. By organizing programs on purpose to enhance interpersonal connections with collaborative physical activity and connect the links to academic supports. These approaches not just align with developmental science but also offer a scalable, affordable way to enhance overall student well-being and academic success.

## 5 Conclusion and recommendations

In conclusion, our results underscore the importance of combined interventions for adolescent development in physical, social, and academic domains. This study demonstrates that physical activity enhances academic engagement not only directly but also through a distinct serial mediational pathway—first by strengthening interpersonal relationships and subsequently by increasing perceived academic support.

While the convergence with recent international research (Odio et al., 2022) is universal, associations with cultural differences in effect sizes do exist. Future intervention research will have to determine whether combined increases in physical activity, social skills training, and academic support will provide synergistic benefits on student outcomes (Magistro et al., 2022). Based on these findings, contemporary physical education programs should have explicit social–emotional learning components (Durlak et al., 2022), emphasize intensive longitudinal designs (Iida et al., 2023), cross-cultural replications (Tao et al., 2022), multi-level intervention studies (Vasilopoulos et al., 2023), and probing digital mediation effects (Twenge, 2019) to prompt academic engagement. This highlights that while core mechanisms may be transferable, direct replication of findings without accounting for contextual factors could limit their generalizability. In terms of reproducibility, future studies should prioritize methodological rigor to ensure consistent outcomes across replications. This underscores that while the core mechanisms may retain cross-context relevance, direct replication of findings without contextual adjustments risks constraining their generalizability. For reproducibility, future research should prioritize methodological precision—standardizing protocols, clarifying measurement parameters, and documenting procedural nuances—to ensure consistent outcomes across replications.

## Data Availability

The raw data supporting the conclusions of this article will be made available by the authors, without undue reservation.
